# Supplementary material for: Structure-guided design of pure orthosteric inhibitors of αIIbβ3 that prevent thrombosis but preserve hemostasis
Source: Nat Commun. 2020 Jan 21;11:398. doi: 10.1038/s41467-019-13928-2 (PMC6972956; doi:10.1038/s41467-019-13928-2)
Supplement: Supplementary file 1 — Supplementary Information [file 41467_2019_13928_MOESM1_ESM.pdf]

Supplementary Information

Structure-guided design of novel orthosteric inhibitors of αIIbβ3 that prevent thrombosis but preserve hemostasis

Brian D. Adair, José L. Alonso, Johannes van Agthoven, Vincent Hayes, Hyun Sook Ahn,
I-Shing Yu, Shu-Wha Lin, Jian-Ping Xiong, Mortimer Poncz,
M. Amin Arnaout

a

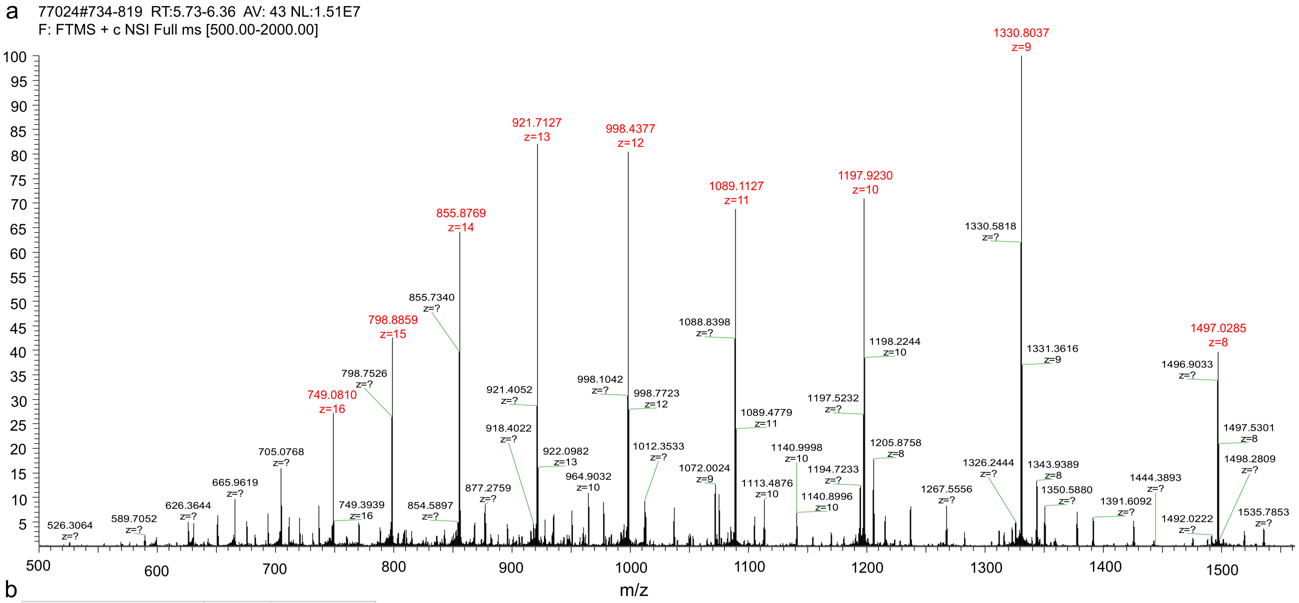

b

Sample : Hr10			
	m/z	Charge	mass
m/z_largest	1497.0285	8	11968.228
m/z_2	1330.8037	9	11968.2333
m/z_3	1197.9230	10	11969.23
m/z_4	1089.1127	11	11969.2397
m/z_5	998.4377	12	11969.2524
m/z_6	921.7127	13	11969.2651
m/z_7	855.8769	14	11968.2766
m/z_8	798.8859	15	11968.2885
m/z_9	749.0810	16	11969.296
Average mass		SD	
11,968.8122		0.528	

Supplementary Figure 1. **Mass spectroscopy analysis of Hr10**. a) The mass spectrum from the intact Hr10 sample. Major peaks are displayed with assigned charges. b) Table of the largest peaks showing the m/z ratios for the larges peaks, the calculated charge and resulting molecular weight. The molecular weight calculated from nine peaks is 119868.9 ± 0.5 (mean ± S.D.) as compared to the calculated molecular weight of the protein lacking the N-terminal Met and with a single L-Har substitution (11,969.3). Peaks corresponding to the protein with Arg at 1493 could not be identified (not shown).

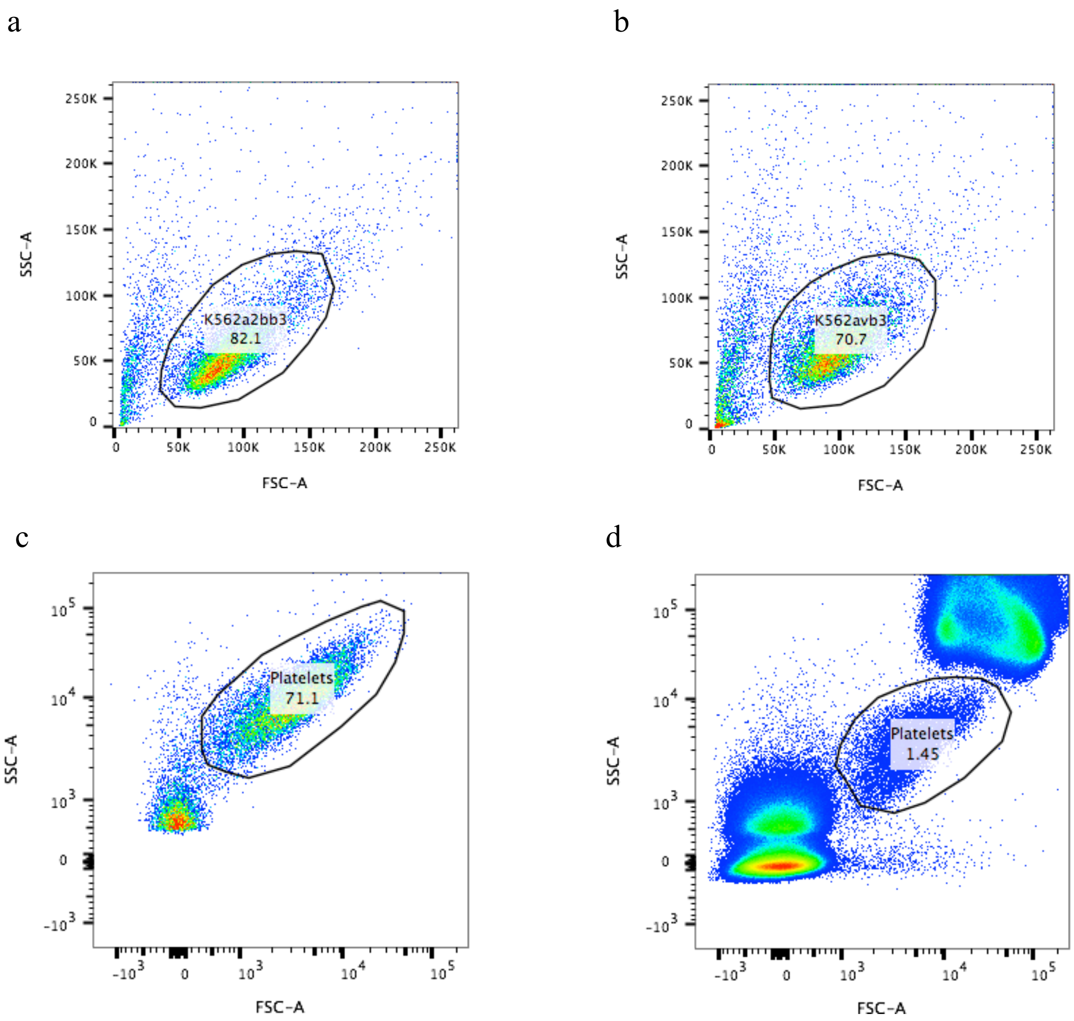

Supplementary Figure 2. Gating strategies used for ligand binding studies. a) Gating strategy for live stable αIIbβ3-K562 cells used in FACS analysis presented in Fig. 2a, 2b *inset*, 2c, Fig.5c, Fig.7c and Fig.8c, d. b) Gating strategy for live stable αVβ3-K562 cells used in FACS analysis presented in Fig. 2b, 2b *inset* and Supplementary Fig.5. c) Gating strategy for fresh human platelets in PRP used in FACS analysis presented in Fig.2d 2d *inset* and Fig.8a. d) Gating strategy for fresh human platelets in whole blood used in FACS analysis presented in Fig. 4f.

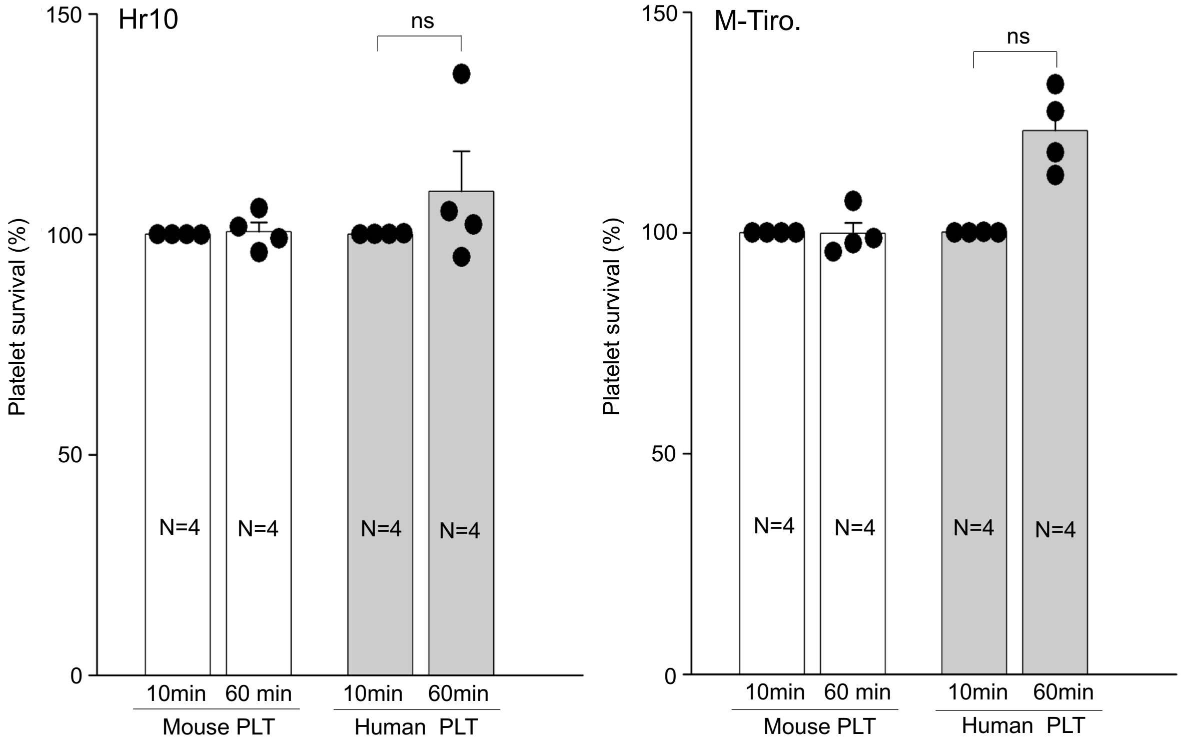

Supplementary Figure 3. Human platelet clearance in humanized mice. Humanized mice infused with human platelets were then injected with PBS, Hr10 or M-Tirofiban and platelet count was checked 10 and 60 minutes after injection (mean $\pm$ S.E; n=4 per group). Mouse platelet counts at 10 min were scaled to 100%. ns, not significant, based on Student two-tailed t-test.

a LCMS (m/z): calcd. for C26H31N3O5, Exact Mass: 465.2; found, m/z = 466 (M+1), 464 (M-1); ^{1}H NMR (400 MHz, CD3OD): δ 8.2 (d, 1H), 7.2 (m, 4H), 7.0-6.8 (m, 4H), 4.7 (m, 1H), 4.0 (t, 2H), 3.3 (m, 2H), 3.1 (m, 2H), 2.9 (m, 2H), 1.9 (m, 2H), 1.7 (m, 2H), 1.5 (m, 3H), 1.3 (m, 4H); ^{13}C NMR (400 MHz, CD3OD): δ 173.7, 161.3, 159.6, 158.1, 148.4, 131.5, 131.4, 129.6, 126.5, 126.2,121.0, 120.6, 117.0, 116.0, 115.7, 115.6, 68.6, 55.5, 45.3 (2C), 37.2, 36.7, 34.8, 30.3, 30.0, 23.9.

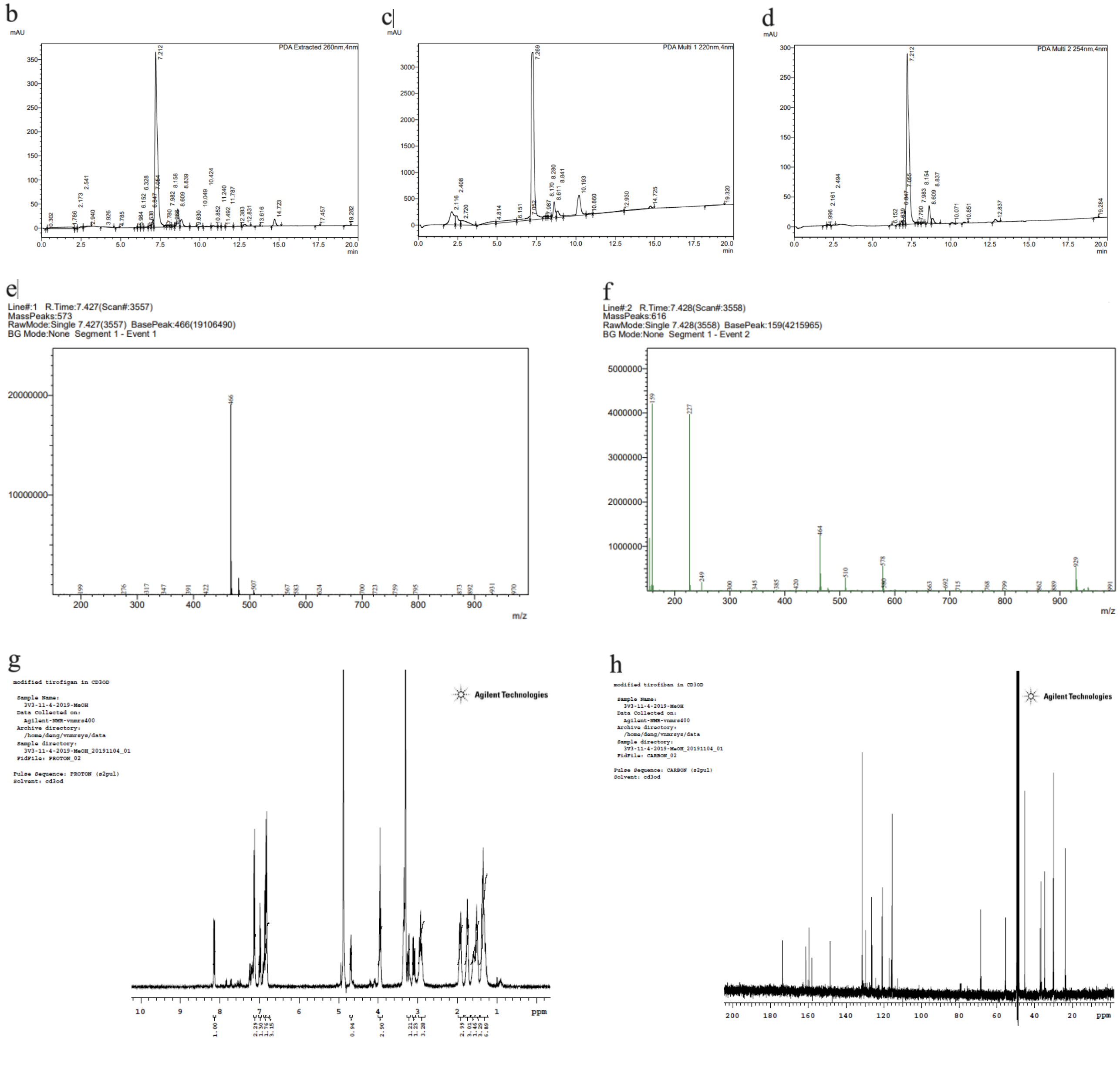

Supplementary Figure 4. Mass spectroscopy analysis of M-Tirofiban. a) M-Tirofiban characterization data. b-d) LC Chromatograms of M-Tirofiban at 260nm (b), 220nm (c) and 254nm (d). e-f) MS spectra of M-Tirofiban, m/z=466 (M+1) (e), and m/z=464 (M-1)(f). g) ^{1}H NMR spectrum of M-Tirofiban. Multiplicity for peaks (s = singlet, d = doublet, dd = double doublet, t = triplet, q = quartet, m = multiplet, br = broad) is shown in parentheses in (a). h) ^{13}C NMR spectrum of M-Tirofiban.

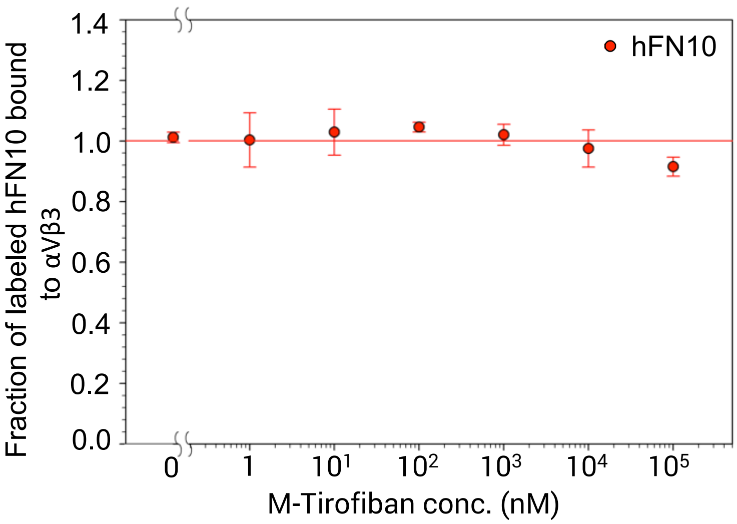

Supplementary Figure 5. **M-Tirofiban does not bind αVβ3**. Graph displaying the residual binding of fluorescently labeled hFN10 to αVβ3-K562 cells in the presence of increasing amounts of unlabeled M-tirofiban. The plot displays the mean and S.E. for three independent experiments along with a line for the mean value. The mean MFI values for each run were used to normalize the data. A two-way Anova to determine the dependence of the FACS run (factor 1) and the M-Tirofiban dose (factor 2) on the MFI for the three experiments indicated no statistically significant dependence ($p=0.331$) on M-Tirofiban dose.

Supplementary Table 1. Data collection and refinement statistics.

Data collection	**αVβ3/Hr10**
PDB Code	6NAJ
Beamline	ID19 at APS
Space group	$P3_221$
Unit cell dimensions (Å, º)	$a=b$=129.7, c=308.2; α=β=90, γ=120
Resolution range (Å)	50-3.1
Wavelength (Å)	0.97932
Total reflections	1,044,981
Unique reflections	55,225 (5,444) *
Completeness	100 (100)
Redundancy	8.2 (8.0)
Molecules in asymmetric unit	1
Average I/σ	24.9 (2.0)
R_{merge} (%)	9.7 (100)
R_{meas} (%)	10.3 (100)
R_{sym} (%)	3.6 (38.8)
Wilson B-factor	59.6
Refinement statistics	
Resolution range (Å)	49.2-3.1
R_{factor} (%)	24.9 (33.9)
R_{free} (%) #	27.4 (38.9)
No. of atoms	13,498
Protein	13,137
Water	4
Mn^{2+}	8
Glc-NAc	349
Average B-factor for all atoms (Å^2)	71.1
Protein	69.4
Ligand/Ion	
Ligand (Hr10)	104.4
Mn^{2+}	53.8
Water	50.5
r.m.s. deviations	
Bond lengths (Å)	0.004
Bond angles (°)	1.03
Ramachandran plot	
Most favored (%)	90.9
Allowed regions (%)	8.7
Outliers (%)	0.4
Clashscore (%)	7.7
Rotamer outliers (%)	2.4

* Values in parentheses are for the highest resolution shell (0.1Å)

R_{free} was calculated with 5% of the data
