# Supplementary material for: Structure-guided design of pure orthosteric inhibitors of αIIbβ3 that prevent thrombosis but preserve hemostasis
Source: Nat Commun. 2020 Jan 21;11:398. doi: 10.1038/s41467-019-13928-2 (PMC6972956; doi:10.1038/s41467-019-13928-2)
Supplement: Supplementary file 3 — Reporting Summary [file 41467_2019_13928_MOESM3_ESM.pdf]

nature research

Corresponding author(s): M. Amin Arnaout

Last updated by author(s): 2019/11/05

Reporting Summary

Nature Research wishes to improve the reproducibility of the work that we publish. This form provides structure for consistency and transparency in reporting. For further information on Nature Research policies, see Authors & Referees and the Editorial Policy Checklist.

Statistics

For all statistical analyses, confirm that the following items are present in the figure legend, table legend, main text, or Methods section.

n/a	Confirmed	
[ ]	[X]	The exact sample size (n) for each experimental group/condition, given as a discrete number and unit of measurement
[ ]	[X]	A statement on whether measurements were taken from distinct samples or whether the same sample was measured repeatedly
[ ]	[X]	The statistical test(s) used AND whether they are one- or two-sided<br>*Only common tests should be described solely by name; describe more complex techniques in the Methods section.*
[ ]	[X]	A description of all covariates tested
[ ]	[X]	A description of any assumptions or corrections, such as tests of normality and adjustment for multiple comparisons
[ ]	[X]	A full description of the statistical parameters including central tendency (e.g. means) or other basic estimates (e.g. regression coefficient) AND variation (e.g. standard deviation) or associated estimates of uncertainty (e.g. confidence intervals)
[ ]	[X]	For null hypothesis testing, the test statistic (e.g. F, t, r) with confidence intervals, effect sizes, degrees of freedom and P value noted<br>*Give P values as exact values whenever suitable.*
[X]	[ ]	For Bayesian analysis, information on the choice of priors and Markov chain Monte Carlo settings
[X]	[ ]	For hierarchical and complex designs, identification of the appropriate level for tests and full reporting of outcomes
[ ]	[X]	Estimates of effect sizes (e.g. Cohen's d, Pearson's r), indicating how they were calculated

Our web collection on statistics for biologists contains articles on many of the points above.

Software and code

Policy information about availability of computer code

Data collection	1. Aggrolink 8: a commercial sotware package for control of the ChronoLog model 700 aggregometer (Havertown, PA).<br>2. Diffraction data was collected at ID-19 of APS, indexed, integrated, scaled by HKL2000 65, solved by molecular replacement in PHASER and refined in Phenix.
Data analysis	SigmaPlot: a commercial software package for scientific graphing and data analysis (Systat Software, San Jose, CA)

For manuscripts utilizing custom algorithms or software that are central to the research but not yet described in published literature, software must be made available to editors/reviewers. We strongly encourage code deposition in a community repository (e.g. GitHub). See the Nature Research guidelines for submitting code & software for further information.

Data

Policy information about availability of data

All manuscripts must include a data availability statement. This statement should provide the following information, where applicable:

- Accession codes, unique identifiers, or web links for publicly available datasets
- A list of figures that have associated raw data
- A description of any restrictions on data availability

All relevant data are included in the paper and/ or its supplementary information files. The source data underlying Figs. 1c-f; 3e-f; 4a, b, c, e; 5c-h, 6a-c and Supplementary Figs 3 and 5 are provided as a Source Data file. The atomic coordinates and structure factors for the reported crystal structure of αVβ3/Hr10 complex have been deposited in the Protein Data Bank (PDB) under the accession code 6NAJ [http://www.rcsb.org/pdb/results/results.do?tabtoshow=Unreleased&qrid=AD6A3C5C], where they can be obtained free of charge.

Field-specific reporting

Please select the one below that is the best fit for your research. If you are not sure, read the appropriate sections before making your selection.

[X] Life sciences [ ] Behavioural & social sciences [ ] Ecological, evolutionary & environmental sciences

For a reference copy of the document with all sections, see nature.com/documents/nr-reporting-summary-flat.pdf

Life sciences study design

All studies must disclose on these points even when the disclosure is negative.

Sample size	Sample size calculations used formulas by La Morte, W. (Ref 73), assuming that hemostasis is preserved in 80% of Hr10 or M-Tirofiban-treated mice but only 5 % of Eptifibatide or Tirofiban-treated mice (projections supported by published reports of similar studies using Eptifibatide and Tirofiban, and the predictive clot retraction data).
Data exclusions	No data were excluded from the analysis of samples.
Replication	All attempts made to replicate samples were successful.
Randomization	Allocation of animals was random.
Blinding	Blinding was not done since one investigator experienced in either the thrombosis or the bleeding humanized mouse models carried out the respective study.

Reporting for specific materials, systems and methods

We require information from authors about some types of materials, experimental systems and methods used in many studies. Here, indicate whether each material, system or method listed is relevant to your study. If you are not sure if a list item applies to your research, read the appropriate section before selecting a response.

Materials & experimental systems

n/a	Involved in the study	
[ ]	[X]	Antibodies
[ ]	[X]	Eukaryotic cell lines
[X]	[ ]	Palaeontology
[ ]	[X]	Animals and other organisms
[ ]	[X]	Human research participants
[X]	[ ]	Clinical data

Methods

n/a	Involved in the study	
[X]	[ ]	ChIP-seq
[ ]	[X]	Flow cytometry
[X]	[ ]	MRI-based neuroimaging

Antibodies

Antibodies used	1. Alexa Fluor 488-conjugated mAb against human CD62P (Santa Cruz). Cat# sc-8419;Clone: CTB201;Lot# H151<br>2. Alexa Fluor 488-conjugated mAb against human CD63 (Santa Cruz).Cat# sc-5275;Clone: MX-49.129.5;Lot# A2517<br>3. Alexa Fluor647-conjugated anti-human CD42b/GP1b mAb (R&D). Cat# FAB4067R;Lot# 1491694<br>4. APC-labeled goat anti-mouse Fc-specific antibody (Jackson). Cat# 115-136-071;Lot# 134469<br>5. Alexa Fluor-488 labeled F(ab')2 fragment of mouse anti-human CD41a (Catalogue#555465) from BD Biosciences.<br>6. Alexa Fluor-647 rat anti-mouse CD41a F(ab')2 (Catalogue#624101) from BD Biosciences.<br>7. AP3 hybridoma (from ATCC, Catalogue#ATCC HB®-242)<br>8. AP5 obtained from Peter Newman, Univ. Wisconsin<br>9. LIBS-1 (obtained from Mark Ginsberg, USCSD)<br>10. PT-25-2 (from Makoto Handa, Keio University, Tokyo, Japan)
Validation	Abs 1-6 above are validated by the manufacturer.<br>7. AP3 (JBC. 2014; 289:23256-63)<br>8,9. AP5, LIBS-1 (ref 24)<br>10. PT-25-2 (Thromb Haemost. 1996;76: 1038-1046)

Eukaryotic cell lines

Policy information about cell lines

Cell line source(s)	K562 cells stably expressing human αVβ3 cells have been previously described. Nat Struct Mol Biol 21, 383-388 (2014)

Cell line source(s)	(ref.24??)<br>K562 cells stably expressing αIIbβ3 were previously described. J Mol Recognit 24, 127-135 (2011)(ref 67????).
Authentication	Original K562 cell were obtained from ATCC.
Mycoplasma contamination	K562 cell line was not tested for Mycoplasma.
Commonly misidentified lines<br>(See ICLAC register)	*Name any commonly misidentified cell lines used in the study and provide a rationale for their use.*

Animals and other organisms

Policy information about studies involving animals; ARRIVE guidelines recommended for reporting animal research

Laboratory animals	NSG mice were used, age 10-12 weeks. Males were used for the cremasteric muscle injury model and female mice used for the bleeding model.
Wild animals	This study did not use wild animals.
Field-collected samples	This study did not involve samples collected in the field.
Ethics oversight	Animal protocol was approved by the Children's Hospital of Philadelphia Internal Review Board in accord with the Helsinki Principles.

Note that full information on the approval of the study protocol must also be provided in the manuscript.

Human research participants

Policy information about studies involving human research participants

Population characteristics	Blood was obtained from healthy donor males and females.
Recruitment	Random healthy volunteers not taking anti-platelet drugs the previous 10 days who agreed to participate in the study were used as donors.
Ethics oversight	Informed written consent was obtained under protocols approved by the Children's Hospital of Philadelphia and Mass General Hospital Internal Review Boards in accord with the Helsinki Principles.

Note that full information on the approval of the study protocol must also be provided in the manuscript.

Flow Cytometry

Plots

Confirm that:

[X] The axis labels state the marker and fluorochrome used (e.g. CD4-FITC).

[X] The axis scales are clearly visible. Include numbers along axes only for bottom left plot of group (a 'group' is an analysis of identical markers).

[ ] All plots are contour plots with outliers or pseudocolor plots.

[X] A numerical value for number of cells or percentage (with statistics) is provided.

Methodology

Sample preparation	K562 or platelets were stained with the primary/secondary antibodies on ice and fixed in 2% paraformaldehyde.
Instrument	FACSCalibur or BD-LSRII flow cytometers (BD Biosciences).
Software	FlowJo software. FACS Data were presented as histograms or graphs based on the calculated MFIs.
Cell population abundance	No sorting was performed. 10,000 cells were counted in each case.
Gating strategy	Representative figures of the gating strategy for FACS experiments is now provided in the Supplementary Figure 2

[X] Tick this box to confirm that a figure exemplifying the gating strategy is provided in the Supplementary Information.
